# Monocyte/lymphocyte ratio is associated with carotid stenosis in ischemic stroke: A retrospective analysis

**DOI:** 10.1002/brb3.1429

**Published:** 2019-09-30

**Authors:** Bo Zuo, Sha Zhu, Xue Meng, Danhua Zhao, Jun Zhang

**Affiliations:** ^1^ Department of Cardiology Cardiovascular Centre Beijing Friendship Hospital Capital Medical University Beijing China; ^2^ Department of Neurology Peking University International Hospital Beijing China; ^3^ Department of Neurology Peking University People's Hospital Beijing China

**Keywords:** atherosclerosis, carotid artery stenosis, inflammation, ischemic stroke, monocyte/lymphocyte ratio

## Abstract

**Background:**

Carotid artery stenosis, mainly caused by carotid atherosclerosis, is related to ischemic stroke. This study was to investigate whether monocyte/lymphocyte ratio (MLR) was associated with increased severity of carotid stenosis in patients with ischemic stroke.

**Methods:**

A total of 395 participants with ischemic stroke were retrospectively analyzed. The severity of carotid stenosis was evaluated by ultrasound examination. Patients were divided into two groups: nonsevere stenosis group and severe stenosis group. Multivariate logistic analysis was used to evaluate risk factors.

**Results:**

A significant correlation was found between MLR and the severity of carotid stenosis in patients with ischemic stroke. MLR was the independent risk factor of carotid stenosis (OR: 9.74, 95% CI: 1.16–81.54). In the ROC curves analysis, a cutoff value of 0.20 for MLR predicted the severity of carotid stenosis with a sensitivity of 80.40% and specificity of 26.40% (ROC area under the curve: 0.598, 95% CI: 0.53–0.67, *p* = .004).

**Conclusion:**

Monocyte/lymphocyte ratio plays important roles in carotid stenosis in patients with ischemic stroke and is an independent risk factor of the severity of carotid stenosis. Therefore, MLR might be considered a potential index in the diagnosis of carotid stenosis in patients with ischemic stroke.

## Graphical abstract

Monocyte/lymphocyte ratio (MLR) plays important roles in carotid stenosis in patients with ischemic stroke and is an independent risk factor of the severity of carotid stenosis. Therefore, MLR might be considered a potential index in the diagnosis of carotid stenosis in patients with ischemic stroke.

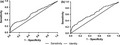

## 1 INTRODUCTION

Ischemic stroke is one of the leading causes of global mortality and illness in the world. With the rapid socioeconomic development and the changes of people's lifestyle, including the increasing working pressure, high fat diet, and lack of exercise, the incidence of ischemic stroke is increasing worldwide year by year (Benjamin et al., 2017). In China, approximately 7 million patients had a stroke and >70% of them had an ischemic stroke. The recurrence rate of ischemic stroke is approximately 8%, and the disability rate is up to 17%, which is the main cause of neurofunctional disability (Chen et al., 2017). Ischemic stroke is associated with a high rate of incidence, recurrence, disability and mortality, which not only affect patients' self‐care ability, but also may result in heavy burdens on their family and society (Chen et al., 2017; Sherzai & Elkind, 2015). Therefore, an early detection of ischemic stroke and its prevention have important practical significance, which may ultimately lead to better health outcomes and reduce the financial burden inherent to diseases associated with a long‐term disability. Carotid artery stenosis is closely related to ischemic stroke (Eckstein et al., 2013; Gupta et al., 2012; Lu & Wang, 2016) and is mainly caused by carotid atherosclerosis (Adams, Bojara, & Schunk, 2018; Liu et al., 2017) because of embolic sequelae as a consequence of underlying atherosclerosis which can incur/cause secondary hemodynamic disturbances. The role of inflammation in the pathogenesis of carotid atherosclerosis has been well established (Borne et al., 2017; Casiglia & Tikhonoff, 2007), and the role of various inflammatory cells and mediators involved in carotid disease and stroke should be well investigated in order to better predict/ identify at‐risk patients (Ammirati, Moroni, Norata, Magnoni, & Camici, 2015; Casiglia & Tikhonoff, 2007; Horne et al., 2005; Woollard & Geissmann, 2010).

The increasing number of leukocyte subtypes, such as eosinophils, neutrophils, and monocytes, is associated with adverse cardiovascular and cerebrovascular events and carotid stenosis (Gijsberts et al., 2017; Hyun et al., 2015; Nunez et al., 2009; Vakili et al., 2017). Monocyte/lymphocyte ratio (MLR, monocyte count/lymphocyte count), neutrophil/lymphocyte ratio (NLR, neutrophil count/lymphocyte count), and platelet/lymphocyte ratio (PLR, platelet count/lymphocyte count) are indicators reflecting the degree of inflammation (Gijsberts et al., 2017; Hyun et al., 2015; Vakili et al., 2017). These parameters or indices are derived from calculations formulated from results obtained from a routine blood analysis; the predictive value of these results obtained from laboratory testing provides a relatively noninvasive and accessible means of obtaining information and profiling individual patients in a time‐sensitive manner in the workup for patient with stroke. In 2015, Hyun et al. (2015) found that NLR may be a clinically significant predictor of the degree of carotid stenosis in patients with ischemic stroke. Inflammation plays critical role in the pathogenesis of carotid atherosclerosis in the pathogenesis of carotid atherosclerosis, and the increase in monocyte number in blood routine is positively related to carotid stenosis (Tanaka et al., 2016). A lymphocyte count decrease was found in cardiovascular and cerebrovascular events, suggesting a poor prognosis (Nunez et al., 2009). Therefore, MLR, closely related to monocytes number and lymphocytes number, as an indicator reflecting the degree of inflammation, its role in carotid artery stenosis in patients with ischemic stroke should be studied at present. Thus, we hypothesized that MLR might be related to the severity of carotid artery stenosis and could be a potential index in the diagnosis of carotid artery stenosis in patients with ischemic stroke. This study mainly aimed at investigating whether MLR was related to the severity of carotid artery stenosis in patients with ischemic stroke. We also investigated the relationship between PLR/NLR and carotid artery stenosis and compared the role of the aforementioned parameters in patients with carotid artery stenosis and ischemic stroke.

## 2 METHODS

### 2.1 Study population

A total number of 395 participants with ischemic stroke who were treated in the neurology department of Peking University People's Hospital from January 2013 to December 2015 were retrospectively analyzed. Ischemic stroke was diagnosed according to the diagnostic criteria of the ischemic stroke revised by the Fourth National Conference on cerebrovascular diseases (Neurology Branch of Chinese Medicine Association, 1996). Exclusion criteria were the following: 3 days after the onset of disease, chronic inflammatory disease, or any systemic infection, severe hepatic and renal insufficiency, tumors, or immunosuppressive disease; patients under immunosuppressants and hormones therapy; and diseases of the blood, digestive or endocrine system. For patients screened for these conditions above, we selected based on medical history, related blood tests, and routine examinations, such as abdominal ultrasound, chest X‐ray, or chest CT. This study was approved by the ethics committee of Peking University People's Hospital, and written informed consent was obtained from all patients.

### 2.2 Data collection

After admission, 10 ml of venous blood were collected after fasting for 10 hr and analyzed for leukocyte count and basic biochemical clinical data, including fasting plasma glucose and uric acid using an automatic blood analyzer. Patients’ demographic data, such as medical history, personal history, and family history, were acquired, while the diagnosis of hypertension and diabetes was determined according to 2010 Chinese Guidelines for the management of hypertension and Chinese Guidelines for the management of type 2 diabetes (2013), respectively (Chinese Diabetes Society, 2014; Liu, 2011).

### 2.3 Carotid ultrasonography

Carotid ultrasonography was carried out by qualified ultrasound technologists, and all protocols were performed according to the Chinese stroke vascular ultrasound examination guidelines. Each common carotid artery, internal carotid artery, external carotid artery, and bulb were examined and recorded for the presence of atherosclerotic plaques in the longitudinal and transverse planes. The severity of carotid artery stenosis was assessed according to the criteria recommended by a multidisciplinary panel of the Society of Radiologists in Ultrasound (Grant et al., 2003). Chinese guidelines for the management of carotid artery stenosis (Vascular Surgery Group, 2017) suggest to re‐examine and monitor the development of carotid artery stenosis in patients with carotid artery stenosis was not <50%. According to this suggestion, thus, stenosis severity was classified into nonsevere stenosis (stenosis < 50%) and severe stenosis (stenosis ≥ 50%) and patients were divided into two groups: nonsevere stenosis group (*n* = 298) and severe stenosis group (*n* = 97) according to this classification.

### 2.4 Statistical analysis

SPSS16.0 software was used for statistical analysis. Continuous variables were expressed as mean ± *SD* or median (interquartile range) when appropriate. Discrete variables were presented as percentages; the intergroup differences were analyzed using variance test for normally distributed measurement data, while rank sum test was used for non‐normally distributed data; the intergroup differences were analyzed using chi‐square test or Fisher's exact test. Univariate logistic regression analysis was performed in all clinical variables, and results with *p* < .05 were considered statistically significant. Variables included in logistic regression analysis were determined based on the results above and professional knowledge with *p* < .05 as statistical significance to comprehensively analyze stenosis risk factors of carotid arteries. ROC curve of MLR was developed to calculate the area under the curve and the 95% confidence interval. Since MLR was mainly used to screen patients with severe carotid stenosis, optimal operating point (OOP) was determined as the cut point to obtain a sensitivity of at least 80% and maximize specificity (Critschristoph, Muenz, & Tu, 2003). Two‐sided difference with *p* < .05 was considered statistically significant.

## 3 RESULTS

Table S1 lists the baseline demographic and biochemical characteristics of the study population. Among the 395 patients, 270 were male, and the mean age was 63.91 ± 11.78 years. The median MLR, NLR, and PLR was 0.25 (0.19–0.32), 2.09 (1.66–2.90), and 108.29 (85.27–135.58) respectively (see details in Table S1). Table S2 shows the correlation between MLR and other inflammatory indexes from blood routine examination, which indicated that MLR could reflect the inflammation degree and should be further studied (see details in Table S2).

The population was divided into two groups according to the MLR median (0.25) in Table 1. Group 1 (MLR ≤ 0.25) included 195 patients (114 males, age 61.74 ± 11.34 years) and group 2 (MLR > 0.25) included 200 patients (156 males, age 66.03 ± 11.84 years). Overall, significant differences between MLR and gender, lymphocyte count, monocyte count, neutrophil count, PLT count, NLR, and PLR were found (*p* < .05). In addition, MLR was significantly correlated with the severity of carotid artery stenosis (37/195 (19%) versus 60/200 (30%), *p* = .011). No statistically significant correlation was found between MLR and other indexes.

**Table 1:** Baseline characteristics of the study population based on MLR level

	MLR ≤ 0.25 (*n* = 195)	MLR > 0.25 (*n* = 200)	*p*
Age	61.74 ± 11.34	66.03 ± 11.84	<.001
Plaque			
High‐density echo	44 (23%)	37 (19%)	.507
Mixed‐echo	132 (68%)	146 (73%)	
Low‐density echo	19 (10%)	17 (9%)	
Male	114 (58%)	156 (78%)	<.001
Previous stroke	47 (24%)	54 (27%)	.509
Smoke	106 (54%)	118 (59%)	.352
HT	133 (68%)	153 (77%)	.065
Hyperlipidemia	51 (26%)	39 (20%)	.115
DM	72 (37%)	91 (46%)	.083
CAD	25 (13%)	24 (12%)	.805
Drink	75 (38%)	88 (44%)	.264
Stenosis	37 (19%)	60 (30%)	.011
Heart rate	76 (70–76)	76 (70–78)	.319
FBG	5.43 (4.79–7.13)	5.43 (4.73–7.14)	.637
WBC	6.50 (5.33–7.58)	6.55 (5.55–7.99)	.237
Lymphocyte	2.11 (1.80–2.42)	1.57 (1.27–1.80)	<.001
Monocyte	0.40 (0.34–0.48)	0.50 (0.42–0.61)	<.001
Neutrophil	3.67 (2.93–4.55)	4.24 (3.37–5.47)	<.001
PLT	203.19 ± 60.00	191.71 ± 53.51	.045
Uric acid	317.04 ± 81.50	318.82 ± 89.98	.905
NLR	1.72 (1.39–2.05)	2.70 (2.09–3.51)	<.001
PLR	93.18 (76.75–114.94)	120.39 (100.71–156.47)	<.001

Abbreviations: CAD, coronary artery disease; DM, diabetes mellitus; FBG, fasting blood‐glucose; HT, hypertension; MLR, monocyte/lymphocyte ratio; NLR, neutrophil/lymphocyte ratio; PLR, platelet/lymphocyte ratio; PLT, platelet; WBC, white blood cell.

**Figure 1:**
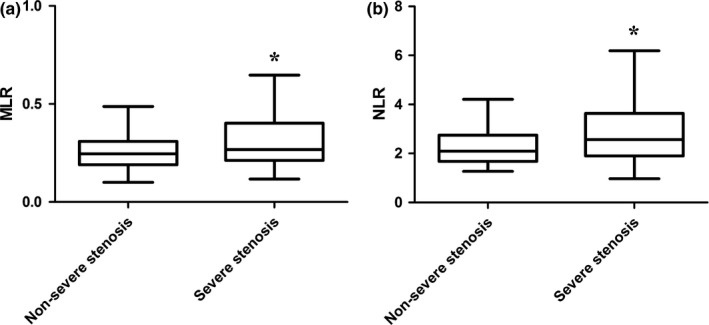
MLR and NLR comparison in patients with ischemic stroke divided by carotid artery stenosis indexes. (a) MLR in patients with ischemic stroke divided by carotid artery stenosis. (b) NLR in patients with ischemic stroke divided by carotid artery stenosis. *versus nonsevere stenosis group, *p* < .05. MLR, monocyte/lymphocyte ratio; NLR, neutrophil/lymphocyte ratio

As shown in Table 2, patients in the severe stenosis group were older and showed more conventional carotid artery stenosis risk factors, including advanced age, male gender, and smoke compared with nonsevere stenosis group. Besides, MLR (0.27 [0.21–0.40] vs. 0.25 [0.19–0.31], *p* = .004) and NLR (2.57 [1.92–3.62] vs. 2.00 [1.59–2.69], *p* < .001) were significantly higher in severe stenosis group compared with nonsevere stenosis group (Figure 1).

**Table 2:** Baseline characteristics of the study population based on stenosis severity

	Nonsevere stenosis group (*n* = 298)	Severe stenosis group (*n* = 97)	*p*
Age	63.23 ± 11.80	65.99 ± 11.53	.044
Plaque			
High‐density echo	77 (26%)	4 (4%)	<.001
Mixed‐echo	193 (65%)	85 (88%)	
Low‐density echo	28 (9%)	8 (8%)	
Male	192 (64%)	78 (80%)	.003
Previous stroke	68 (23%)	33 (34%)	.031
Smoke	160 (54%)	64 (66%)	.034
HT	221 (74%)	65 (67%)	.171
Hyperlipidemia	73 (24%)	17 (18%)	.155
DM	121 (4%)	42 (43%)	.640
CAD	35 (12%)	14 (14%)	.485
Drink	112 (38%)	51 (53%)	.009
Heart rate	76 (70–76)	76 (68–78)	.699
FBG	5.34 (4.74–7.06)	5.88 (4.89–7.20)	.186
WBC	6.25 (5.27–7.56)	7.18 (6.11–8.61)	<.001
Lymphocyte	1.81 (1.52–2.21)	1.75 (1.31–2.15)	.112
Monocyte	0.44 (0.37–0.53)	0.48 (0.38–0.59)	.018
Neutrophil	3.70 (3.02–4.75)	4.53 (3.67–6.00)	<.001
PLT	196.16 ± 50.79	201.11 ± 73.13	.459
Uric acid	314.98 ± 84.74	327.02 ± 88.77	.230
NLR	2.00 (1.59–2.69)	2.57 (1.92–3.62)	<.001
PLR	104.20 (84.26–132.08)	113.61 (91.89–143.11)	.052
MLR	0.25 (0.19–0.31)	0.27 (0.21–0.40)	.004

Abbreviations: CAD, coronary artery disease; DM, diabetes mellitus; FBG, fasting blood‐glucose; HT, hypertension; MLR, monocyte/lymphocyte ratio; NLR, neutrophil/lymphocyte ratio; PLR, platelet/lymphocyte ratio; PLT, platelet; WBC, white blood cell.

**Figure 2:**
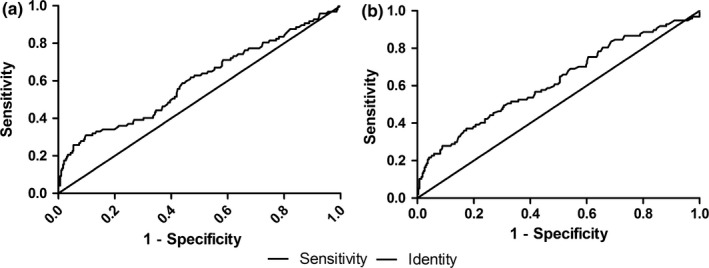
Diagnostic accuracy of MLR and NLR on carotid artery stenosis in patients with ischemic stroke by ROC curve. (a) Diagnostic accuracy of MLR on carotid artery stenosis in patients with ischemic stroke was analyzed by ROC curve. (b) Diagnostic accuracy of NLR on carotid artery stenosis in patients with ischemic stroke was analyzed by ROC curve. MLR, monocyte/lymphocyte ratio; NLR, neutrophil/lymphocyte ratio

ROC curve was used to analyze MLR and NLR efficiency in detecting the severity of carotid artery stenosis. A cutoff value of 0.20 for MLR predicted the severity of carotid artery stenosis, with a sensitivity of 80.40% and specificity of 26.40% (ROC area under the curve: 0.598, 95% CI: 0.53–0.67, *p* = .004; Figure 2a); A cutoff value of 1.81 for NLR predicted the severity of carotid artery stenosis, with a sensitivity of 80.40% and specificity of 40.30% (ROC area under the curve: 0.656, 95% CI: 0.59–0.72, *p* < .001; Figure 2b). Thus, both MLR and NLR might be helpful for a correct diagnosis of the severity of carotid artery stenosis.

Multivariate logistic analysis was used to assess the risk factors in the severity of carotid artery stenosis in patients with ischemic stroke. The regression result in Table 3 demonstrated that MLR was the independent risk factor of the severity of carotid artery stenosis (OR: 9.74, 95% CI: 1.16–81.54), together with neutrophil count, plaque feature, and hypertension, while NLR was not. Finally, MLR, Plaque, Neutrophil, HT, MLR*Plaque, MLR*Neutrophil, MLR*HT, Plaque*Neutrophil, Plaque*HT, Neutrophil*HT was included in the logistic regression model to test potential interactions, which shows that the interactions between MLR and Plaque, Neutrophil, HT on the severity of carotid artery stenosis was significant (*p* < .05) (Table 4).

**Table 3:** Multivariate logistic regression analysis to assess severity of carotid stenosis

Variable	*B*	Wald	*p* Value	OR	95% CI
Age	0.023	3.629	.057	1.023	0.999–1.048
Drink	0.481	3.008	.083	1.617	0.939–2.785
MLR	2.277	4.411	.036	9.744	1.164–81.544
Neutrophil	0.298	11.650	.001	1.348	1.135–1.599
Plaque		14.582	.001		
Plaque (1)	−1.651	5.965	.015	0.192	0.051–0.722
Plaque (2)	0.396	0.767	.381	1.486	0.612–3.604
HT	−0.580	3.996	.046	0.560	0.317–0.989
Previous stroke	0.502	2.923	.087	1.653	0.929–2.939

Note

Plaque (1): High‐density echo plaque compared to low‐density echo plaque. Plaque (2): Mixed‐echo plaque compared to low‐density echo plaque.

Abbreviations: CI, confidential interval; HT, hypertension; MLR, monocyte to lymphocyte ratio; OR, odds ratio.

**Table 4:** Potential interactions between risk factors on severity of carotid stenosis

Variable	*B*	Wald	*p* Value	OR	95% CI
MLR*HT	−1.844	3.88	.049	0.158	0.025–0.991
MLR*Neutrophil	0.868	11.816	.001	2.382	1.452–3.907
MLR*Plaque	2.866	10.831	.001	17.563	3.187–96.787
MLR	−5.645	5.446	.020	0.004	0.000–0.405

Abbreviations: CI, confidential interval; HT, hypertension; MLR, monocyte/lymphocyte ratio; OR, odds ratio.

## 4 DISCUSSION

Monocyte/lymphocyte ratio, calculated from a blood routine analysis, is inexpensive, simple, safe, and noninvasive. Our study confirmed its potential as a good inflammatory index and its strong correlation with white blood cells, neutrophils, and NLR. Therefore, the clinical utility of these indices could prove informative in expanding our knowledge on the biochemical profiles of patients with TIA or stroke. High MLR values indicate a poor prognosis of multiple malignancies. Indeed, MLR was initially applied as a predictor of lymphoma, nasopharyngeal carcinoma, and bladder, lung, and breast cancer (Li et al., 2012; Ni et al., 2014; Temraz et al., 2014; Teng, Zhang, Zhang, Zhang, & Li, 2016). In addition, MLR was valuable in the diagnosis of infectious diseases such as malaria, AIDS, and tuberculosis (Naranbhai, Hill, et al., 2014; Naranbhai, Kim, et al., 2014; Warimwe et al., 2013). Therefore, we focused our attention on MLR to evaluate its potential as a diagnostic marker. Li et al found that MLR was closely related to the degree of coronary plaque lesions in patients with non‐ST‐elevated myocardial infarction (Ji et al., 2017), suggesting that MLR may be an indicator of atherosclerosis.

To the best of our knowledge, no reports are available regarding the relationship between MLR and the degree of carotid stenosis. Therefore, this study mainly focused on and demonstrated the relationship between MLR and carotid artery stenosis in ischemic stroke patients. Our study found that MLR had a positive correlation with the degree of carotid artery stenosis in ischemic stroke patients, and it was an independent factor, suggesting that MLR might be a potential diagnostic marker for the severity of carotid artery stenosis.

Carotid artery stenosis development and occurrence is due to a long‐term chronic inflammatory process. Lymphocytes and mononuclear cells are the main inflammatory cells in the body and actively participate in the formation of carotid atherosclerotic plaque and carotid artery stenosis (Ammirati et al., 2015; Woollard & Geissmann, 2010). In the plaque early‐stage formation, together with smoking and hypertension, the migration of monocytes into the vessel wall and their differentiation lead to the formation of a stable plaque, consequent vessel wall thickening, and subsequent vascular stenosis. With the progression of the lesion, after the injury of the endothelial cells, the monocyte/macrophage can destabilize the plaque under specific incentive stimulation, causing thrombosis and embolus, and eventually leading to the occurrence of adverse cardiovascular and cerebrovascular events. Therefore, the aggregation and activation of monocytes and their derived macrophages are actively participating in the onset of atherosclerosis (Casiglia & Tikhonoff, 2007; Incalcaterra et al., 2013). The lymphocyte levels represent the level of cellular and humoral immunity in vivo, which is inversely proportional to the progress of atherosclerosis. In particular, regulatory T lymphocytes have significant anti‐inflammatory effects. High monocyte counts and low lymphocyte counts were independent risk factors of cerebrovascular disease (Nunez et al., 2009; Tanaka et al., 2016). Therefore, MLR, such as the integration of monocytes and lymphocytes into a single index, might be a potential indicator of the severity of the atherosclerotic plaque. The results of this study confirmed that MLR was an independent risk factor for carotid artery stenosis, which was consistent with our expectations. Compared with NLR, MLR may more significantly influence on carotid artery stenosis.

Horne et al. (2005) found that NLR was significantly and positively associated with the maximum carotid intima‐media thickening value in men in a retrospective study with 252 patients. NLR may be a predictor of the severity of carotid stenosis in patients with ischemic stroke, which indicates that NLR, as well as MLR, is really worthy of studying, as other biomarkers assessing carotid stenosis are useful, but costly and complicated. This study showed that as well as NLR, MLR could be considered a potential clinical index. Except the reason above that MLR might reflect well on the progress of atherosclerosis and the severity of the atherosclerotic plaque, the difference on sample size, race of study population, and the variables included may also lead to the difference of the two studies.

The pathological mechanism might be related to the following processes: (a) the monocytes in the patients with high MLR level might produce a large number of inflammatory mediators to accelerate the inflammatory response, thereby promoting lipid deposition and plaque formation in the vascular walls. (b) Inflammatory cytokines released from inflammatory cells in patients with high MLR level might promote excessive proliferation of smooth muscle cells, thus promoting atherosclerosis (Gupta et al., 2012; Li et al., 2012).

Based on ROC curve analysis and a cutoff value of 0.20, MLR predicted carotid artery stenosis with a sensitivity of 80.40% and specificity of 26.40%. In method section above, we mentioned that as MLR was used to screen patients with severe carotid stenosis, OOP was determined as the cut point to obtain a sensitivity of at least 80% and maximize specificity. In order to meet the requirement on sensitivity, we sacrifice the level of specificity in order to emphasize the significance of the sensitivity. As ensuring the high sensitivity, we could target patients needed to be concerned more exactly, reduce the scope of potential population greatly, and reduce the cost markedly. Therefore, although the specificity of MLR was relatively low, a preliminary screening of the presence or formation of carotid stenosis through MLR might reduce medical costs especially critical in poorer areas, as MLR is obtained by blood routine test, which is necessary for almost all patients with ischemic stroke. Almost no additional cost is added by MLR in most cases. In addition, early intervention through the combination of MLR and carotid artery ultrasound examination might be helpful to screen carotid stenosis effectively. It is important to point out that MLR, as a single index, could not solve all problems. The poor specificity could be made up by other examinations after preliminary screening by MLR.

In this study, we found that MLR was the independent risk factor of the severity of carotid artery stenosis, while NLR was not. As we aforementioned, lymphocyte and mononuclear cells are the main inflammatory cells in the formation of carotid atherosclerotic plaque and carotid artery stenosis. Therefore, MLR, combined with lymphocyte count and monocyte count, might reflect the inflammatory state of carotid stenosis. MLR could be a potential index to predict the progression of carotid stenosis in patient with ischemic stroke.

Our study has some limitations. First, only MLR baseline measurements were collected, and MLR values over time were not obtained. More information should be collected in future to get more reliable conclusion. Second, this study is a retrospective single‐center study, a prospective, and multi‐center cross‐sectional research should be designed in future. Third, the relationship between MLR and the incidence of MACCE (major adverse cardiac and cerebral vascular events) and death was not explored in this study. Thus, further study is needed to be performed in the future.

In conclusion, our study found that MLR was significantly correlated with the severity of carotid artery stenosis and was an independent risk factor. In addition, the combination of blood routine examination and carotid artery ultrasound examination has a potential clinical value in the diagnosis of carotid artery stenosis and being potentially useful to screen carotid stenosis in patients with carotid artery stenosis.

## CONFLICT OF INTEREST

None declared.

## Data Availability

The data used to support the findings of this study are available from the corresponding author upon request.
