# Supplementary material for: Monocyte/lymphocyte ratio is associated with carotid stenosis in ischemic stroke: A retrospective analysis
Source: Brain Behav. 2019 Sep 30;9(10):e01429. doi: 10.1002/brb3.1429 (PMC6790304; doi:10.1002/brb3.1429)
Supplement: Supplementary file 1 [file BRB3-9-e01429-s001.docx]

**Supplemental Table 1. Baseline Characteristics of the Study Population**

| Variable | n=395 |
| --- | --- |
| Age | 63.91±11.78 |
| Plaque |  |
| High - density echo | 81(21%) |
| Mixed-echo | 278(70%) |
| Low - density echo | 36(9%) |
| Male | 270(68%) |
| Previous Stroke | 101(26%) |
| Smoke | 224(57%) |
| HT | 286(72%) |
| [Hyperlipoidemia](http://www.baidu.com/link?url=HM_oKn5u1BkgiEbXQV4A23bJflirXMO9R2lVZ2_AxRSPFMpuoyK6geg-1COwdXcAjkB9L2VOdZclEy-Dka4_V0xIEBkf5o1Dq2-41hlTL1urOsng3FfBtUyfBcWNpk73) | 90(23%) |
| DM | 163(41%) |
| CAD | 49(12%) |
| Drink | 163(41%) |
| Stenosis | 97(25%) |
| Heart Rate | 76 (70-78) |
| FBG | 5.43(4.76 -7.15) |
| WBC | 6.51 (5.46 -7.86) |
| Lymphocyte | 1.80(1.46 -2.20) |
| Monocyte | 0.45(0.37 -0.54) |
| Neutrophil | 3.85(3.11 -5.00) |
| PLT | 197.38±57.03 |
| Uric acid | 317.94±85.79 |
| MLR | 0.25(0.19-0.32) |
| NLR | 2.09 (1.66 -2.90) |
| PLR | 108.29 (85.27 -135.58) |

HT , hypertension; DM, diabetes mellitus; CAD,coronary artery disease; FBG, fasting blood-glucose; WBC: [white blood cell](http://www.baidu.com/link?url=ksXNIqzg-EWVxmqkZuy7ldntFJxbSxJlZh_UBS24IDrn-N1gEGSIEiNNpmY5E51BMmUlyi7OSj1DbeaV5JTkxwMl1mSIY0obfvQ0rgJ1eu1n_yLF-yCHA0TIK7zKG5of); MLR: Monocyte to lymphocyte ratio ; PLT: Platelet; NLR, neutrophil to lymphocyte ratio; PLR, Platelet to lymphocyte ratio.

**Supplemental Table 2. The correlation between MLR and other inflammatory index in blood routine**

|  | WBC | Neutrophil | NLR | P |
| --- | --- | --- | --- | --- |
| r | 0.235 | 0.397 | 0.691 | 0.549 |
| P | <0.001 | <0.001 | <0.001 | <0.001 |

Spearman test was used.

WBC: [white blood cell](http://www.baidu.com/link?url=ksXNIqzg-EWVxmqkZuy7ldntFJxbSxJlZh_UBS24IDrn-N1gEGSIEiNNpmY5E51BMmUlyi7OSj1DbeaV5JTkxwMl1mSIY0obfvQ0rgJ1eu1n_yLF-yCHA0TIK7zKG5of); NLR, neutrophil to lymphocyte ratio; PLR, Platelet to lymphocyte ratio.
